# Development of a system to support warfarin dose decisions using deep neural networks

**DOI:** 10.1038/s41598-021-94305-2

**Published:** 2021-07-20

**Authors:** Heemoon Lee, Hyun Joo Kim, Hyoung Woo Chang, Dong Jung Kim, Jonghoon Mo, Ji-Eon Kim

**Affiliations:** 1 Department of Thoracic and Cardiovascular Surgery, Sejong General Hospital, Bucheon-si, Gyeonggi-do Republic of Korea; 2 Department of Anesthesiology and Pain Medicine, Anesthesia and Pain Research Institute, Severance Hospital, Yonsei University College of Medicine, Seoul, Republic of Korea; 3 Department of Thoracic and Cardiovascular Surgery, Seoul National University Bundang Hospital, 82 Gumi-ro 173 beon-gil, Bundang-gu, Seongnam-si, Gyeonggi-do 13620 Republic of Korea; 4 Kakao Enterprise, Gyeonggi-do, Republic of Korea; 5 Medical Convergence Research Center, Wonkwang University Hospital, Jeollabuk-do, Republic of Korea

**Keywords:** Cardiology, Cardiovascular biology, Computer science, Scientific data

## Abstract

The first aim of this study was to develop a prothrombin time international normalized ratio (PT INR) prediction model. The second aim was to develop a warfarin maintenance dose decision support system as a precise warfarin dosing platform. Data of 19,719 inpatients from three institutions was analyzed. The PT INR prediction algorithm included dense and recurrent neural networks, and was designed to predict the 5th-day PT INR from data of days 1–4. Data from patients in one hospital (n = 22,314) was used to train the algorithm which was tested with the datasets from the other two hospitals (n = 12,673). The performance of 5th-day PT INR prediction was compared with 2000 predictions made by 10 expert physicians. A generator of individualized warfarin dose-PT INR tables which simulated the repeated administration of varying doses of warfarin was developed based on the prediction model. The algorithm outperformed humans with accuracy terms of within ± 0.3 of the actual value (machine learning algorithm: 10,650/12,673 cases (84.0%), expert physicians: 1647/2000 cases (81.9%), *P* = 0.014). In the individualized warfarin dose-PT INR tables generated by the algorithm, the 8th-day PT INR predictions were within 0.3 of actual value in 450/842 cases (53.4%). An artificial intelligence-based warfarin dosing algorithm using a recurrent neural network outperformed expert physicians in predicting future PT INRs. An individualized warfarin dose-PT INR table generator which was constructed based on this algorithm was acceptable.

## Introduction

Warfarin is a potent blood thinner and was first introduced as a rodenticide in 1948. Warfarin was approved for medical use in the United States in 1954. Currently, warfarin is one of the most frequently prescribed drugs for patients with cardiovascular disease. There are various indicators for warfarin medication, including deep vein thrombosis, pulmonary thromboembolism, atrial fibrillation, valvular heart disease, and artificial heart valves^1^.

The effect of warfarin is measured with a blood coagulation test which generates the prothrombin time international normalized ratio (PT INR). A healthy subject without a coagulation disorder would show a PT INR level of approximately 1.0. Each patient taking warfarin has a specific target PT INR range (e.g., 1.5–2.0, 1.8–2.3, 2.0–2.5, or 2.5–3.0)^1^. Excessive anticoagulation can lead to life-threatening bleeding while insufficient anticoagulation can result in unwanted thrombosis. However, the warfarin dose required to achieve the target PT INR range is determined with several days of trial and error, which means that hospitalization and daily blood sampling may be necessary^2,3^. The pharmacokinetics of warfarin depends on multiple factors, including drug interactions, genetic variations, and diet^4–6^. Due to the complexity of warfarin’s pharmacokinetics, warfarin’s influence on the PT INR is frequently delayed by several days. Therefore, physicians still have difficulty determining the optimal dose of warfarin^7^. Prolonged hospitalization and frequent outpatient clinic visits are commonly required to adjust warfarin doses. Despite the inconvenience of warfarin dose adjustments, many of the indications for anticoagulation still require the administration of warfarin because there is no oral anticoagulant that can substitute for it^8^. For example, a recent randomized controlled trial that compared the efficacy of an oral direct thrombin inhibitor and warfarin was stopped early due to an excess of thromboembolic and bleeding events in the direct thrombin inhibitor group^9,10^.

There have been several studies that used mathematical modeling or machine learning to assist in warfarin dosing^11–18^. Previous research has mainly depended on genetic or drug interaction information to increase the accuracy of warfarin dose prediction. However, these methods are insufficiently precise and genetic analysis is impractical due to its cost. Each patient has their own metabolism with its own specific pharmacokinetics and pharmacodynamics, so predicting warfarin doses based only on constant variables such as sex, age, race, body mass index, genetic test results is clearly limited in reliability.

This study had two goals. The first goal was to develop an individualized PT INR prediction model that learns from patients’ previous responses to warfarin. The second goal was to develop an individualized clinical support system for setting warfarin maintenance doses that used the PT INR prediction model, which is a precision warfarin dosing platform that predicts PT INRs. The decision support system was developed in a way that it can be easily used in clinical practice.

## Methods

### Data preparation

**Figure 1:**
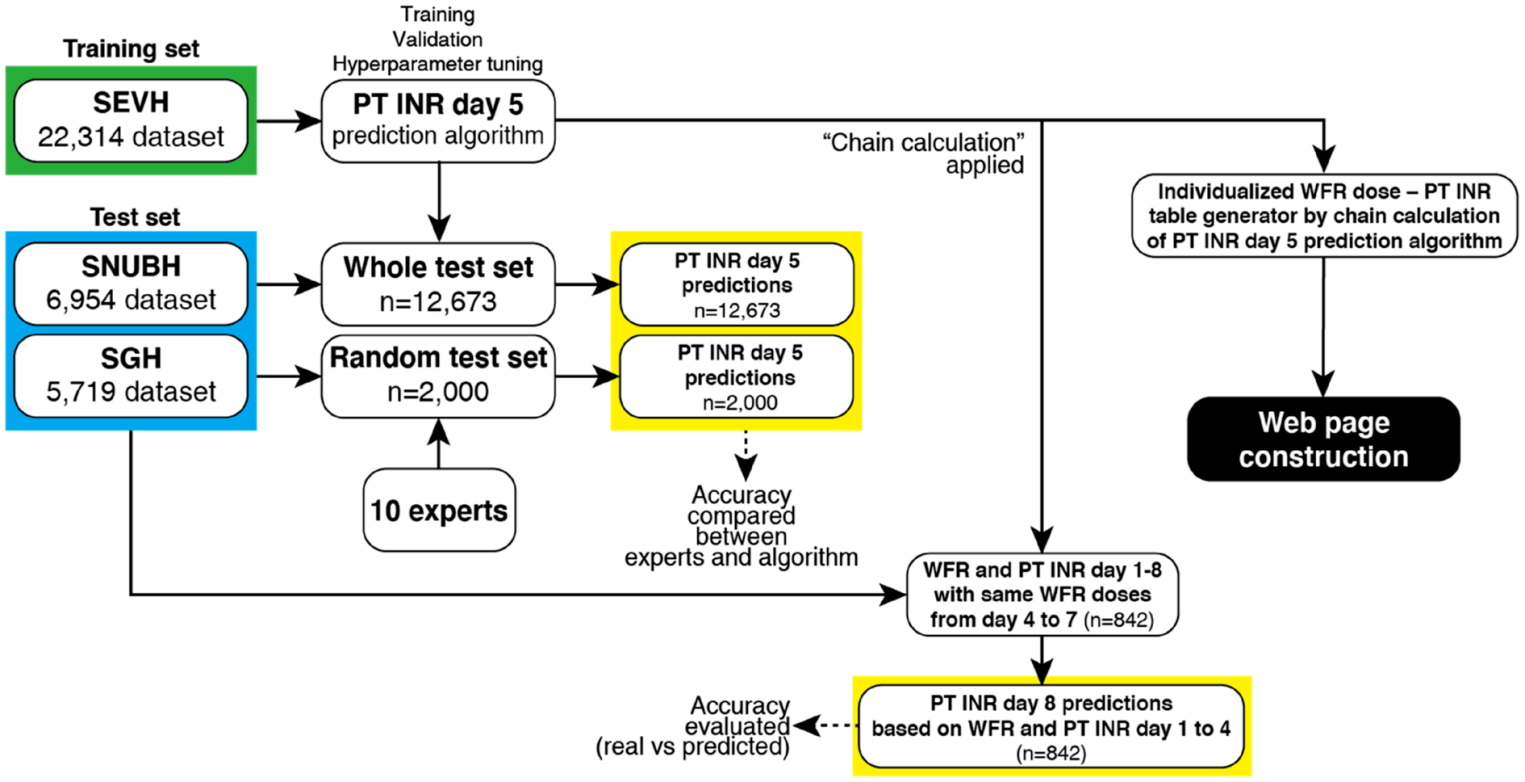
Complete architecture of the whole study. *SEVH* Severance Hospital, *SNUBH* Seoul National University Bundang Hospital, *SGH* Sejong General Hospital, *PT INR* prothrombin time international normalized ratio, *WFR* warfarin.

**Figure 2:**
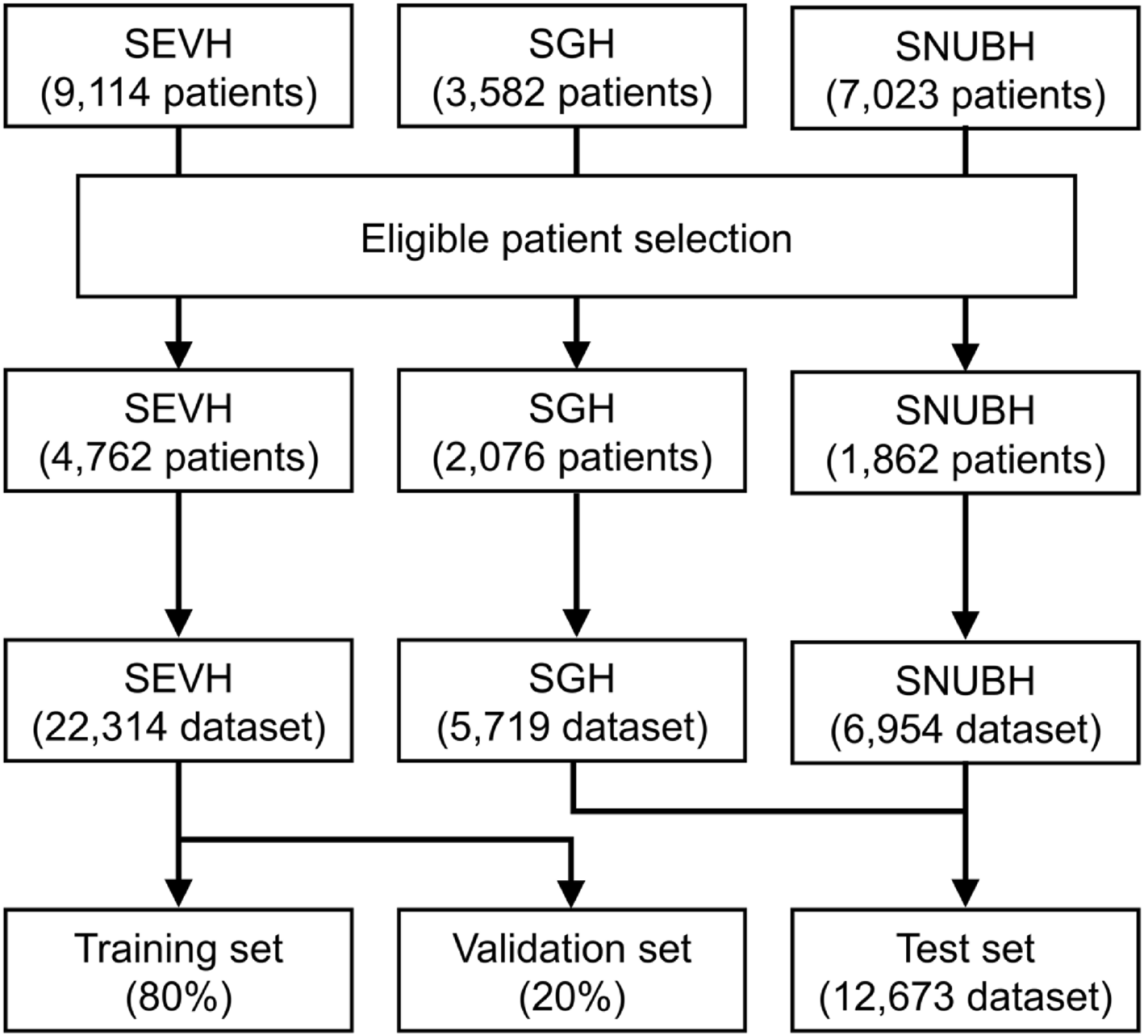
A diagram of how the data was prepared. *SEVH* Severance Hospital, *SGH* Sejong General Hospital, *SNUBH* Seoul National University Bundang Hospital.

Complete architecture of the study is shown in Fig. 1. Only retrospectively collected data was used in this study. Patients included in the study had to be inpatients, taking warfarin, and be at least 18 years old. Baseline patient characteristics were sex, age, body weight, and height. PT INR values and warfarin dose information were collected. Data were collected via the electronic health information systems of Severance Hospital (SEVH, from 2008 to 2018), Sejong General Hospital (SGH, from 2010 to 2018), and Seoul National University Bundang Hospital (SNUBH, from 2003 to 2018) (Fig. 2). SEVH and SNUBH are tertiary referral hospitals and SGH is a specialized cardiovascular intervention and surgery institute.

Data was pre-processed using R 3.6.1 (R Foundation for Statistical Computing, Vienna, Austria). The machine learning algorithm was developed and tested with Python 3.5.6 (Anaconda Inc., Austin, TX, USA), including the Keras 2.2.2 and Tensorflow 1.10.0 libraries. Categorical variables are expressed as the value with the percentage in parentheses while continuous variables are expressed as mean ± standard deviation.

**Figure 3:**
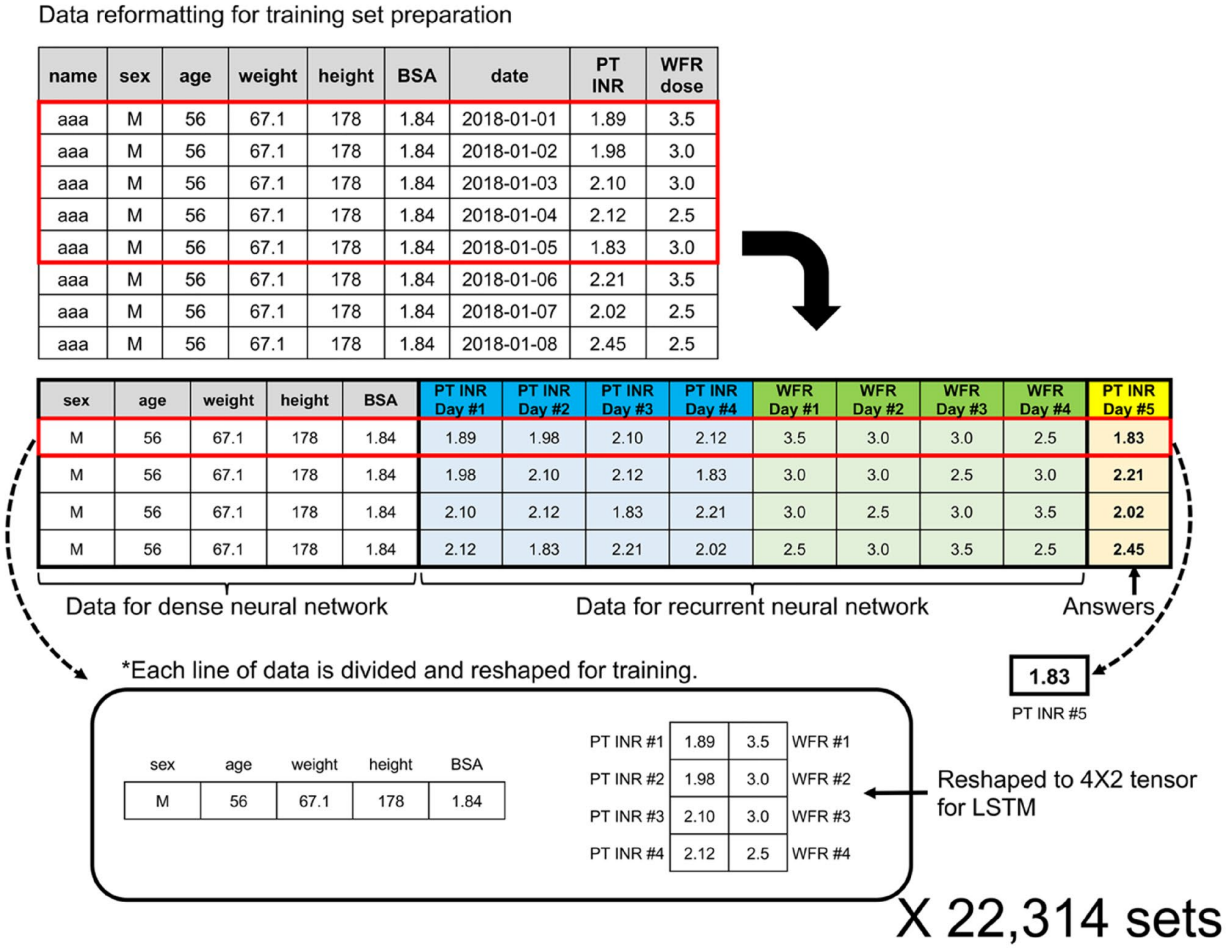
Datasets prepared for the dense and recurrent neural networks (RNN). Raw data were reformatted into data for the dense neural network, recurrent neural network, and correct answers. The standardization process is not shown here to avoid complexity. *BSA* body surface area, *PT INR* prothrombin time international normalized ratio, *WFR* warfarin, *LSTM* long short-term memory.

The pre-processing of the data is summarized in Fig. 3. During pre-processing, outliers or obviously unreliable data were excluded, including observations from patients with weight < 35 kg or > 120 kg, height < 130 cm or > 220 cm, patients who took two or three doses of warfarin per day, PT INRs > 10.0, and daily warfarin doses > 20 mg (see S1a of Supplementary Appendix for inclusion and exclusion criteria). Body surface area (BSA) was calculated using the Mosteller formula^19^. Raw data were checked line by line. To avoid inconsistency or inaccuracy in warfarin doses, we checked the nursing record of whether the drug was taken by the patient very carefully, excluding the doses that were not actually given to the patients. Only data for 5 sequential days were converted into a single-line format using automated coding (see S1b of Supplementary Appendix for dataset baseline characteristics).

### The 5th-day PT INR prediction model

**Figure 4:**
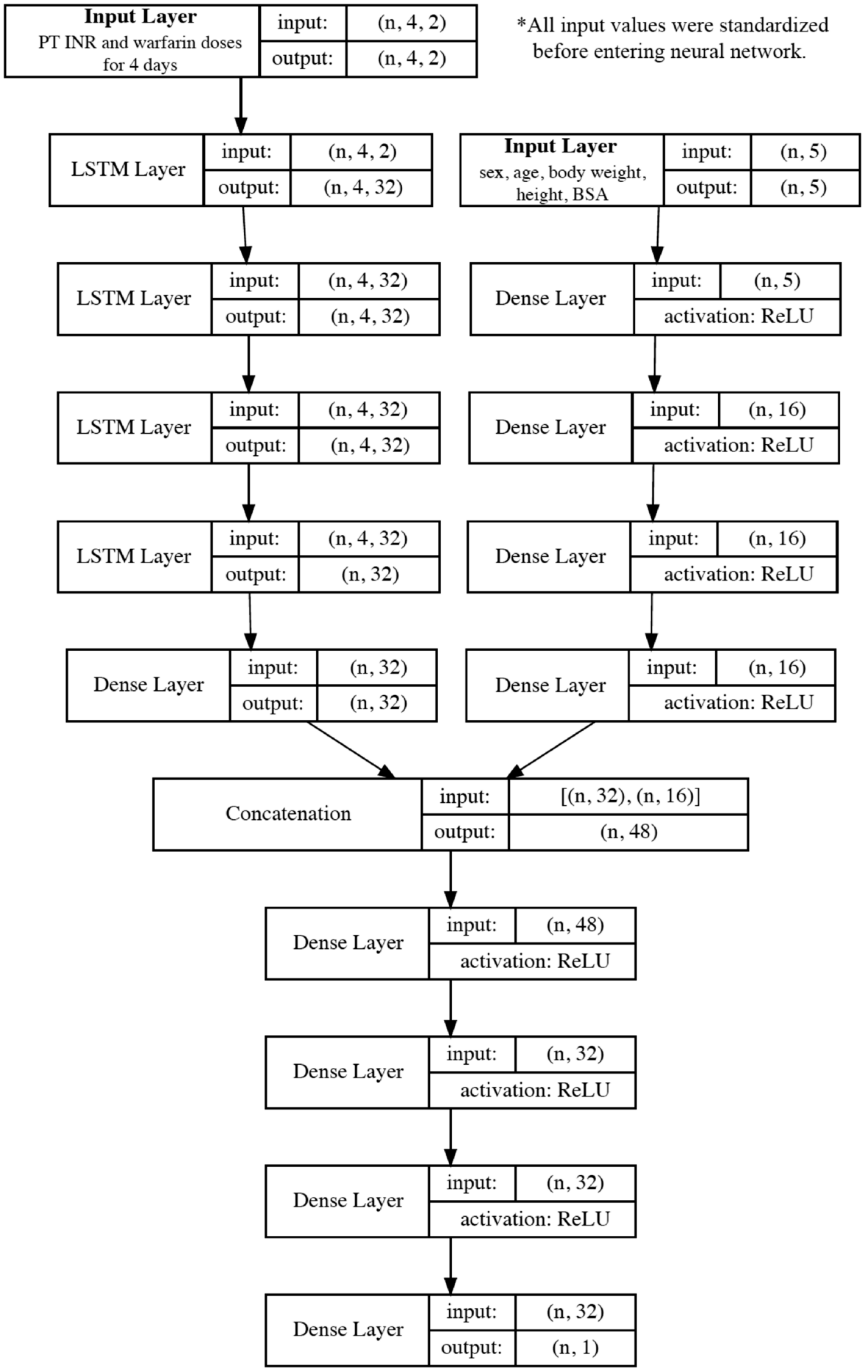
Structure of the 5th-day prothrombin time international normalized ratio (PT INR) prediction model. Five basic variables enter the dense neural network layers and time-series variables (PT INRs and warfarin doses) enter the long-short term memory (LSTM) layers. The parameters from both networks are concatenated and pass through dense neural network layers. Finally, the network produces a single prediction for the 5th-day PT INR. *BSA* body surface area, *WFR* warfarin.

The structure of the 5th-day PT INR prediction model is shown in Fig. 4. All variables were standardized to have a mean of 0 and a standard deviation of 1. Then sex, age, weight, height, and BSA were put into layers of a dense neural network. The time-series variables, namely the PT INR and warfarin dose on days 1–4 were reshaped to 4 × 2 tensors and put into layers of a recurrent neural network with long short-term memory (LSTM). The parameters of the dense neural network and recurrent neural network were concatenated, and the parameters were passed through layers of the dense neural network. Finally, the model produced a single value for the predicted 5th-day PT INR. Rectified linear unit (ReLU) activation functions were used in the dense neural network. Mean absolute error was used as the loss and the Adam optimizer was used for parameter tuning. The model was trained and validated with the SEVH dataset and was tested with the SGH and SNUBH datasets. Training epochs were determined based on the point at which overfitting began which was defined as the constant increase in validation loss (the validation set was set to 20% of the training set). As there was no improvement in mean absolute error when we added more than 5 layers in LSTM, we used 5 layers in LSTM to avoid overfitting. For node numbers, we tested 16, 32, 64, 128, and 256 nodes in each layer, and 32 nodes were chosen because a higher number of nodes resulted in overfitting. In the dense layers for baseline patient characteristics, we limited the number of nodes to 16 because there were only five input variables in the dense layers. In the dense layers for concatenated tensors, we fixed the node numbers at 32 and limited the number of layers to 4 after tests based on manual adjustment. The detailed model structure is presented in Sect. 2 of the Supplementary Appendix.

### Comparison with expert physicians

The performance of this 5th-day PT INR prediction model was evaluated by comparing it to the performance of expert physicians. A total of 2000 questions were prepared from the SGH and SNUBH datasets (see S3 of Supplementary Appendix for an example of questionnaire). Ten expert physicians with at least 3 years of experience prescribing warfarin made predictions about the questions, with each physician answering 200. They predicted the 5th-day PT INR based on the same information that the machine learning algorithm received. A successful prediction was defined as being less than 0.3 different from the actual value^2^. The ups and downs of PT INR in the ± 0.3 range is generally tolerated because changes in this range rarely affect the clinical course. The number of very inaccurate predictions, defined as a difference of > 0.5 from the actual value, were compared.

### Individualized dose PT INR table generator

**Figure 5:**
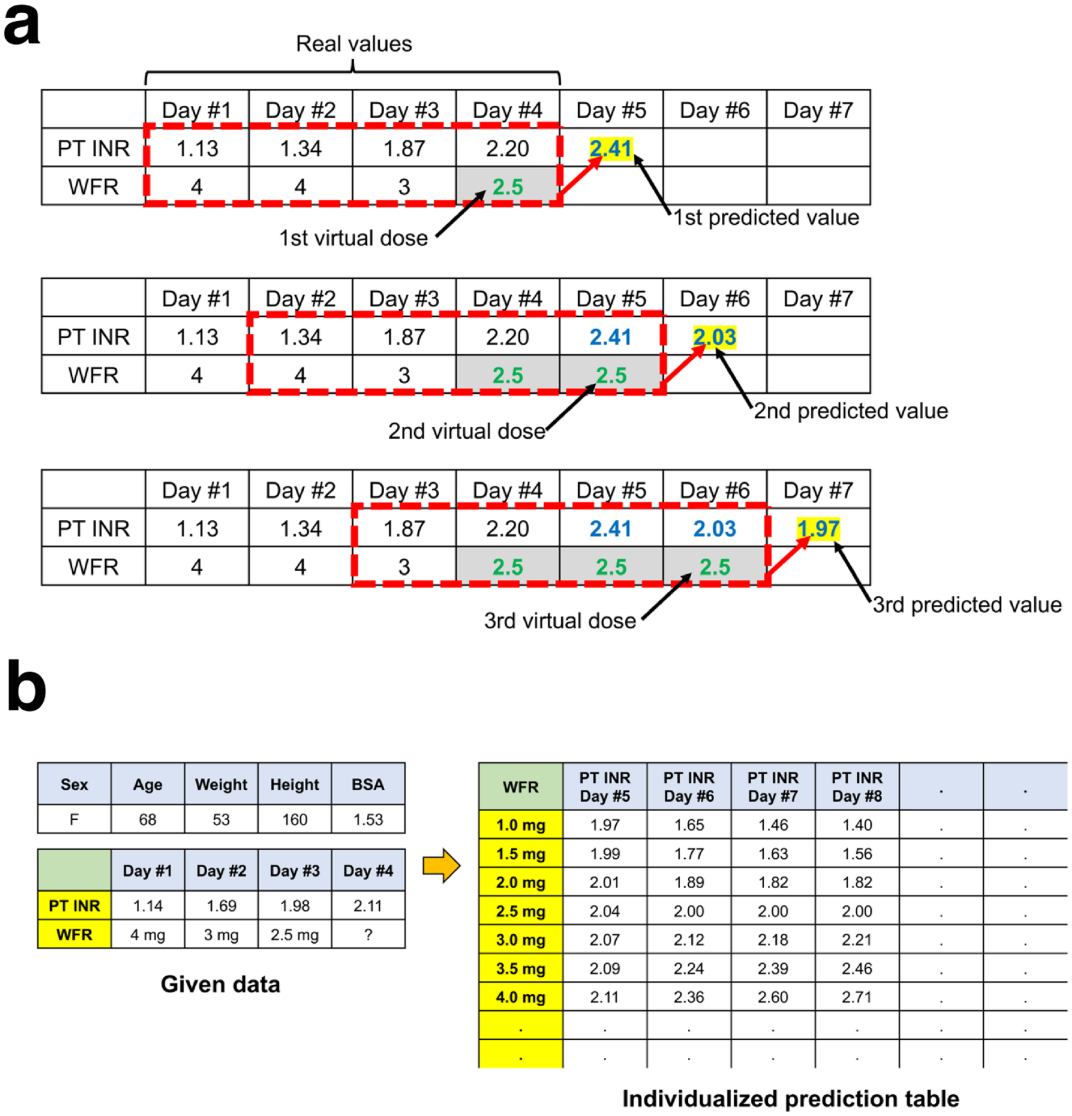
(**a**) A diagram showing how chain calculations are conducted. The chain calculation behind PT INR predictions were based on the assumption that fixed doses of warfarin were administered. The PT INRs on days 1–4 and the warfarin doses administered on days 1–3 were real values. An assumed warfarin dose for day 4 was added. With data from days 1–4, the 5th-day PT INR can be predicted. Another virtual warfarin dose for day 5 was added. With data from days 2–5, the 6th-day PT INR can be predicted. (**b**) An example of an individualized warfarin dose-PT INR table. The chain calculation can be conducted for any fixed dose of warfarin. Therefore, the algorithm can generate individualized warfarin dose-PT INR prediction tables. Physicians can refer to them when determining the warfarin dose. *BSA* body surface area, *PT INR* prothrombin time international normalized ratio, *WFR* warfarin.

After improving the performance of the 5th-day PT INR prediction model as much as possible, an individualized dose-PT INR table generator was developed. PT INR predictions for varying doses can be made using chain calculations with virtual warfarin doses. The individualized dose-PT INR tables could be generated from days 1–4 data (Fig. 5, and see S4 of Supplementary Appendix for step-by-step explanation). The predicted 8th-day PT INR was based on data from days 1–4 and fixed doses of warfarin from days 4–7. Therefore, patient data of 8 sequential days in which doses were fixed from day 4 to 7 was selected to evaluate the accuracy of the 8th-day PT INRs predicted by chain calculation (see S5 of Supplementary Appendix).

### Ethics approval, consent to participate

The institutional review board (IRB) of the three participating hospitals [Severance Hospital (SEVH), Sejong General Hospital (SGH), and Seoul National University Bundang Hospital (SNUBH)] approved this study (SEVH IRB No. 4-2019-0291, SGH IRB No. 2020-0152, SNUBH IRB No. B-1809/490-101). Patient consent was waived by IRBs of all three participating hospitals due to the retrospective nature of the study. The study was conducted in accordance with the Declaration of Helsinki and the Harmonized Tripartite Guideline for Good Clinical Practice from the International Conference on Harmonization.

## Results

Data were collected from a total of 19,719 patients from three hospitals (Severance Hospital (SEVH): n = 9114, Sejong General Hospital (SGH): n = 3582, Seoul National University Bundang Hospital (SNUBH): n = 7023). After preprocessing the data, data from 8700 patients was included in the analysis (SEVH: n = 4762, SGH: n = 2076, SNUBH: n = 1862). Training and test datasets for 5 sequential days consisted of baseline patient information (sex, age, body weight, height, and BSA), the PT INRs and warfarin doses for 4 days, and the actual 5th-day PT INR. The training dataset from SEVH had 22,314 samples and test dataset from SGH and SNUBH had 12,673 samples (SGH: 5719, SNUBH: 6954) (Fig. 2).

### Machine learning model predictions compared to expert physician predictions

**Figure 6:**
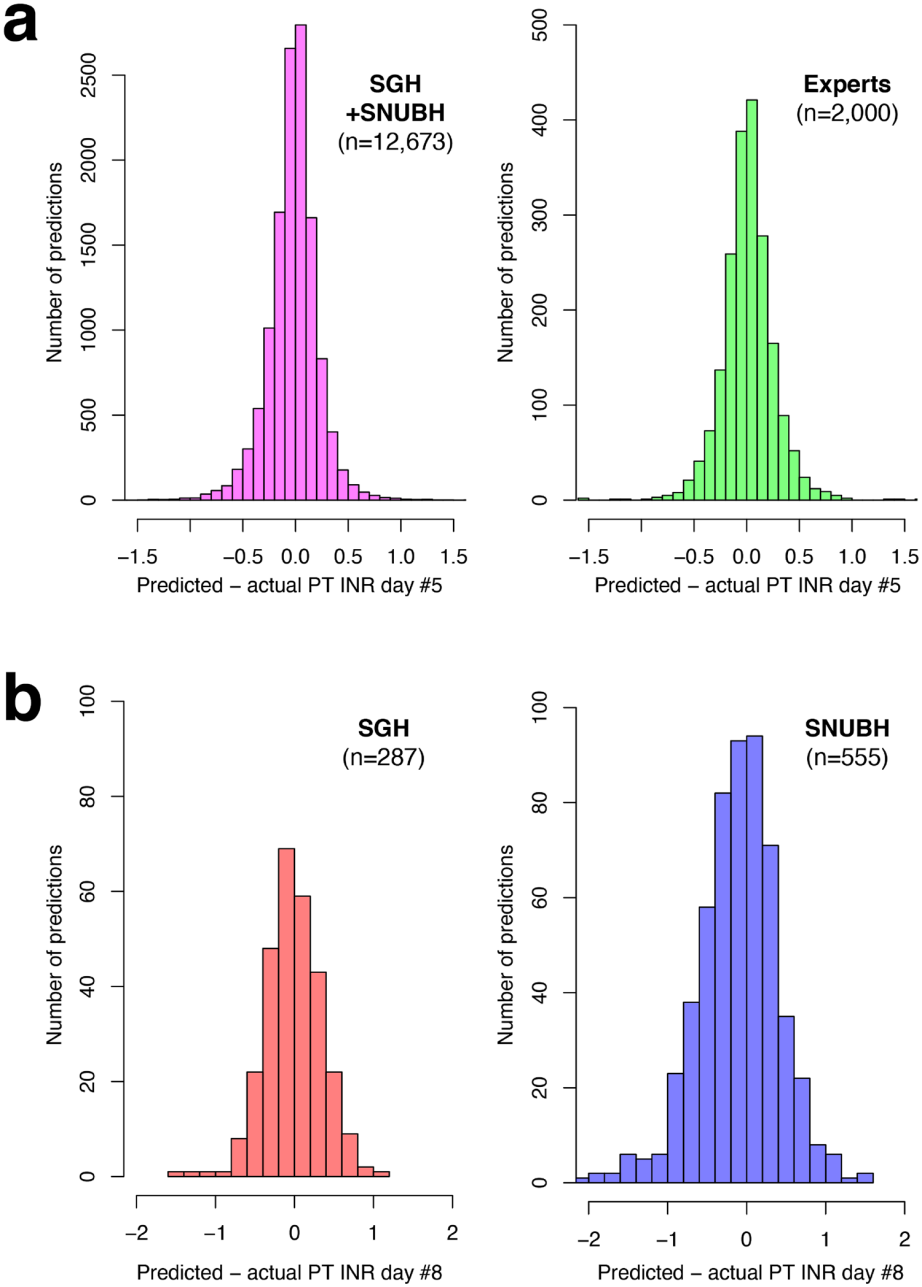
Distribution differences between predicted and actual prothrombin time international normalized ratios (PT INR). (**a**) The predicted 5th-day PT INR based on data from days 1–4. (**b**) The predicted 8th-day PT INR was based on data from days 1–4 and the assumption that fixed doses of warfarin were administered. *SGH* Sejong General Hospital, *SNUBH* Seoul National University Bundang Hospital.

The performance metrics of the machine learning model and expert physicians are compared in Table 1 (see S6 of Supplementary Appendix for performance in each hospital). A histogram of the differences between predicted and actual 5th-day PT INRs is shown in Fig. 6a. The predictions made by the machine learning model were within 0.3 of the actual value in 84.0% of cases (10,650/12,673). This performance was statistically significantly better than that of the 10 expert physicians whose predictions were within 0.3 of the actual value in 81.9% of cases (1647/2000) (*P* = 0.014). The predictions made by the machine learning model were more than 0.5 away from the actual value in 4.8% of cases (605/12673) while those made by the physicians were more than 0.5 away from the actual value in 4.7% of cases (93/2000) (*P* = 0.822).

**Table 1:** The performance of the machine learning algorithm and expert physicians in predicting the 5th-day prothrombin time international normalized ratio (PT INR).

Accuracy	SGH + SNUBH (n = 12,673)	Expert physicians (n = 2000)	P-value
Within 0.2 of the actual value	8806 (69.5%)	1320 (66.0%)	0.002
Within 0.25 of the actual value	9867 (77.9%)	1493 (74.7%)	0.001
Within 0.3 of the actual value	10,650 (84.0%)	1637 (81.9%)	0.014
Outside 0.5 of the actual value	605 (4.8%)	93 (4.7%)	0.822
Outside 1.0 of the actual value	44 (0.4%)	7 (0.4%)	> 0.999
|Predicted value – actual value|(absolute error)	0.170 ± 0.170	0.179 ± 0.174	0.034

The predictions were based on data from days 1–4.

### Dose-PT INR table generator development and evaluation

A table generator that provides PT INR predictions based on virtual administrations of fixed doses of warfarin was developed based on the machine learning algorithm that predicts the 5th-day PT INR (Fig. 5). This warfarin dose-PT INR table generator predicted PT INRs from days 5–8 for warfarin doses of between 1.0 and 8.0 mg in 0.5-mg increments. This table was generated under the assumption that fixed warfarin doses were administered from day 4 to 7. The table showed a gradual stabilization of PT INR from day 4 to 7 with minimal variation (convergence). The converged PT INR values were proportional to the fixed warfarin dose.

The predicted 8th-day PT INR based on the data of days 1–4 were compared with actual data in which the same dose of warfarin was given from day 4 to 7. The table predicted an 8th-day PT INR that was within 0.3 of the actual value in 62.0% of cases (178/287) for the SGH dataset and 49.0% of cases (272/555) for the SNUBH dataset (Table 2, Fig. 6B).

**Table 2:** Prediction of 8th-day prothrombin time international normalized ratio (PT INR) based on data from days 1–4 and assuming that a fixed dose of warfarin was administered from day 4 to 7.

Accuracy	SGH dataset (n = 287)	SNUBH dataset (n = 555)	Total (n = 842)
Within 0.2 of the actual value	126 (43.9%)	187 (33.7%)	313 (37.2%)
Within 0.25 of the actual value	156 (54.4%)	233 (42.0%)	389 (46.2%)
Within 0.3 of the actual value	178 (62.0%)	272 (49.0%)	450 (53.4%)
More than 0.5 away from the actual value	38 (13.2%)	154 (27.4%)	192 (22.8%)
More than 1.0 away from the actual value	4 (1.4%)	31 (5.6%)	35 (4.1%)
|Predicted – actual value|(absolute error)	0.276 ± 0.225	0.396 ± 0.336	0.355 ± 0.308

These predictions were compared to real data for patients who were administered the same dose of warfarin from day 4 to 7.

## Discussion

There are various warfarin dose suggestion models that use artificial intelligence (AI)^17,20,21^ However, these models are all limited in that they were constructed to predict optimum initial starting or maintenance warfarin doses using only cross-sectional data. However, this approach does not reflect the actual human decision-making process. In reality, physicians examine patterns in PT INRs and warfarin doses to determine the optimum steady-state warfarin dose which will maintain the PT INR in the target range when administered repeatedly. Therefore, this study was conducted to develop a model that mimics the human decision-making process. However, training a machine learning model to predict the optimum warfarin dose would be inappropriate because the dose would be an independent variable while the PT INR would be a dependent variable. The optimum warfarin dose is determined when repeated fixed doses of warfarin maintain the PT INR in the target range. However, each patient has their own target range and there is little actual data about repeated administration of fixed warfarin doses. This lack of data makes the reliability of an optimum warfarin dose questionable. Therefore, the model developed in this study was designed to predict future PT INRs based on previous patient data.

The machine learning algorithm developed in this study examined the pattern of changes in PT INRs to determine how it was influenced by warfarin doses over the course of 4 days to predict the 5th-day PT INR as accurately as possible. LSTM, a type of recurrent neural network, was used to discover patterns in sequential PT INR-dose data. LSTM draws on past data when making calculations. LSTM is widely used for machine learning on time-series data, such as natural language processes. The algorithm developed in this study was trained to adapt to individual pharmacokinetic and pharmacodynamic characteristics by examining how fluctuations in a person’s PT INR correlated with their warfarin doses over the 4 days. Several other studies divided patients according to dose ranges and developed a separate algorithm for each and so could not cover a wide dose range^11,17,21^. However, the algorithm developed in this study does not require patients to be classified by dose because it was trained on the whole warfarin dose range and PT INR responses. Theoretically, patients would not need to be classified according to race or ethnicity either if training data includes such characteristics.

Before performing the chain calculation, which was the main interest of this study, the predictions of the 5th-day PT INR had to be verified to be substantially accurate. The accuracy of this prediction is very important because any inaccuracies may be amplified during chain calculations. Therefore, the accuracy of the predictions produced by this study’s algorithm and expert physicians with at least 3 years of warfarin prescription experience were compared. Ten expert physicians made predictions about a total of 2000 questions that were drawn from the machine learning test dataset. The physicians commented that, in clinical practice, they try to determine the optimum warfarin dose, not predict the next-day PT INR, so they are not used to predicting PT INR of the next day. Interestingly, the expert physicians were slightly, but statistically significantly, less accurate than the machine learning algorithm. This difference was smaller than was hypothesized.

The algorithm developed in this study examines data from the preceding 4 days to predict the 5th-day PT INR. Therefore, a chain calculation which is conducted by putting in ‘virtual’ future warfarin dose was possible (Fig. 5). For example, this algorithm could predict the PT INRs of days 5–8 based on repeated fixed doses of warfarin administered on days 4–7. It is possible to vary the input doses, so individualized warfarin dose-PT INR tables can be generated. As in the real world, the predicted PT INR values converged on a static value on days 5–8 under the assumption that a repeated fixed dose of medication is administered. In order to evaluate the accuracy of the predicted 8th-day PT INR based on the data from days 1–4, it was assumed that a fixed dose of warfarin is administered on days 4–7. Then the data used in this study was searched to find data from 8 sequential days in which the same warfarin dose was administered from days 4–7. The accuracy of the 8th-day PT INR by chain calculation was lower than that of the predicted 5th-day PT INR. However, approximately 50% of the predictions were still within 0.3 of the actual value. This level of accuracy was similar to the accuracy of pharmacogenetic algorithms in other studies. However, unlike those algorithms, the algorithm in this study did not require the input of genetic information and did not have a limited target range that cannot be changed^22,23^.

Physicians can refer to the dosing tables generated by the algorithm developed in this study as an aide in determining a patient’s optimum warfarin dose. Using this table will likely reduce fluctuations in warfarin doses by helping physicians more precisely determine the target PT INR range and more accurately selecting warfarin doses from the beginning of administration. An interesting finding in this study was that the accuracy of the predicted 8th-day PT INR differed by hospital that the data came from. The baseline characteristics of datasets from each hospital were similar, but the chain calculation may have amplified differences between the SGH and SNUBH datasets.

The machine learning algorithm developed in this study could be more accurate if it is trained with more variables, such as whether the patient is taking other drugs that can interact with warfarin. However, training it with these extra variables might make it impractical to use the model because of the complexity of the data input process. Therefore, this study was conducted to develop an algorithm that included clinically essential variables that the physicians depend on while making warfarin dose decisions. Clinicians around the world can run this algorithm on their own data through the website aiwarfarin.org.

This study had several limitations. The algorithm was not prospectively tested yet. Its accuracy must be improved before it is used in a prospective trial. Another limitation is that the algorithm was not trained on data which included missed administrations, represented as 0 mg doses. This study also did not account for the addition or discontinuation of other drugs that can interact with warfarin. PT INRs are influenced by a number of factors, so the algorithm’s prediction accuracy may ultimately not be able to exceed a certain level.

## Conclusion

A machine learning algorithm using a recurrent neural network outperformed expert physicians in predicting PT INRs. The individualized warfarin dose-PT INR table generator which was developed based on this algorithm was substantially accurate. If this table generator is integrated in the health information system, it can help physicians reduce errors in warfarin prescription. A prospective study must be conducted to validate the efficacy of this warfarin dose decision platform.

## Supplementary Information

Supplementary Information.

## Data Availability

Data are available upon reasonable request.
