# Supplementary material for: Development of a system to support warfarin dose decisions using deep neural networks
Source: Sci Rep. 2021 Jul 20;11:14745. doi: 10.1038/s41598-021-94305-2 (PMC8292496; doi:10.1038/s41598-021-94305-2)

Supplementary Appendix

for the manuscript entitled

"Development of a system to support warfarin dose decisions using deep neural networks"

Heemoon Lee,[1] Hyun Joo Kim,[2] Hyoung Woo Chang,[3]

Dong Jung Kim,[2] Jonghoon Mo,[4] Ji-Eon Kim[5]

[1] Department of Thoracic and Cardiovascular Surgery, Sejong General Hospital, Gyeonggi-do, Republic of Korea

[2] Department of Anesthesiology and Pain Medicine, Anesthesia and Pain Research Institute, Yonsei University College of Medicine, Seoul, Republic of Korea

[3] Department of Thoracic and Cardiovascular Surgery, Seoul National University Bundang Hospital, Gyeonggi-do, Republic of Korea

[4] Kakao Enterprise, Gyeonggi-do, Republic of Korea

[5] Medical Convergence Research Center, Wonkwang University Hospital, Jeollabuk-do, Republic of Korea

Table of Contents

Section 1. Data characteristics

S1a. Inclusion and exclusion criteria

S1b. Dataset baseline characteristics

Section 2. Algorithm for predicting the 5th-day PT INR and its structure

S2. Tensorflow graph of the model

Section 3. A part of an example question sheet

S3. A part of sample question sheet given to expert physicians

Section 4. Methods of 'chain calculation' in detail

S4. The process of filling up the individualized does-PT INR table

Section 5. Comparison of performance in predicting the 8th-day PT INR

S5. Preparing a comparable dataset from the test dataset

Section 6. Model performance in each hospital

S6. The performance of PT INR Day #5 prediction model in each hospital dataset

Section 1. Data characteristics

S1a. Inclusion and exclusion criteria

**Inclusion criteria**
(1) Inpatient<br>(2) Adult (age: 19–95 years)<br>(3) Administered warfarin daily and have their prothrombin time international normalized ratio (PT INR) checked on at least 5 sequential days
**Exclusion criteria**
(1) Warfarin dose > 20 mg/day<br>(2) PT INR > 10.0<br>(3) Body weight < 35 kg or > 120 kg<br>(4) Height < 130 cm or > 220 cm

S1b. Dataset baseline characteristics. Abbreviations: SEVH, Severance Hospital; SGH, Sejong General Hospital; SNUBH, Seoul National University Bundang Hospital; BSA, body surface area; PT INR, prothrombin time international normalized ratio.

Variable	SEVH dataset (n = 22,314)	SGH dataset (n = 5,719)	SNUBH dataset (n = 6,954)
**Age (years)**	63.2 ± 15.3	62.0 ± 14.0	66.1 ± 14.5
**Sex (male)**	10,820 (48.5%)	2,748 (48.1%)	3,587 (51.6%)
**Weight (kg)**	60.8 ± 12.2	61.4 ± 12.6	61.5 ± 11.8
**Height (cm)**	161.6 ± 9.5	160.8 ± 10.1	161.7 ± 9.4
**BSA (m^2)**	1.65 ± 0.20	1.65 ± 0.21	1.66 ± 0.19
**PT INR**	1.89 ± 0.59	1.81 ± 0.52	1.99 ± 0.52
**Warfarin (mg)**	2.93 ± 1.42	3.01 ± 1.36	2.97 ± 1.68

Section 2. Algorithm for predicting the 5th-day PT INR and its structure

S2. Tensorflow graph of the model.

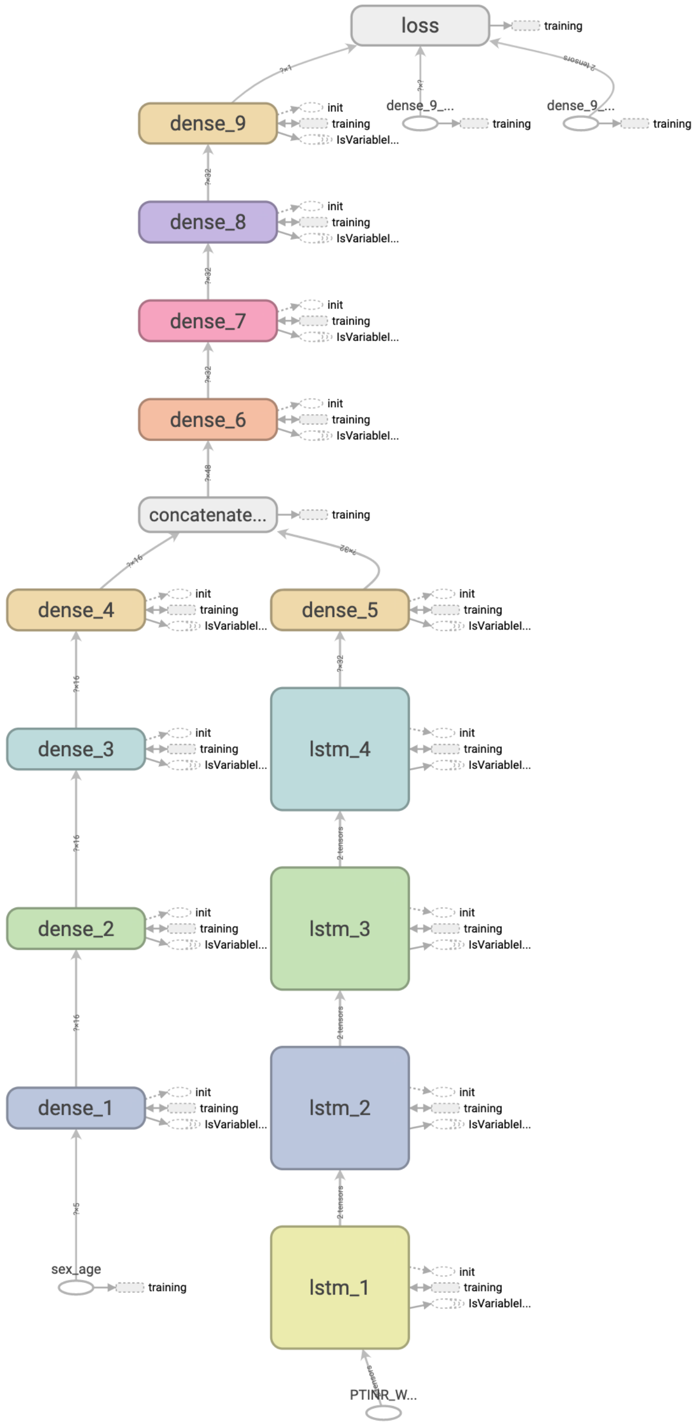

Section 3. A part of an example question sheet

S3. A part of sample question sheet given to expert physicians.

Problem No.	sex	age	bwt	ht	BSA	day	PTINR	WFR	Your answer (PTINR Day_5)
303	F	84	55.4	154	1.54	Day_1	1.82	2.5	
						Day_2	2.27	2	
						Day_3	2.42	1.5	
						Day_4	2.65	1	
						Day_5			?
304	F	60	65	155.9	1.68	Day_1	1.05	5	
						Day_2	1.24	5	
						Day_3	1.31	5	
						Day_4	1.43	5	
						Day_5			?
305	F	49	62.3	167	1.7	Day_1	1.18	4	
						Day_2	1.15	3	
						Day_3	1.27	3	
						Day_4	1.66	1	
						Day_5			?
306	M	64	62	161	1.67	Day_1	1.27	4	
						Day_2	2.2	1	
						Day_3	2.14	2.5	
						Day_4	1.63	3	
						Day_5			?
307	F	30	51	154	1.48	Day_1	1.84	1	
						Day_2	1.73	2	
						Day_3	1.56	2.5	
						Day_4	1.47	3	
						Day_5			?

Section 4. Methods of 'chain calculation' in detail

S4. The process of filling up the individualized does-PT INR table

The table will be constructed to show estimated PT INRs for virtual dose of warfarin.
We hypothesize administration of 'fixed repeated dose' warfarin for clinical practicability.

Sex	Age	Weight	Height	BSA
F	68	53	160	1.53

	Day #1	Day #2	Day #3	Day #4
PT INR	1.14	1.69	1.98	2.11
WFR	4 mg	3 mg	2.5 mg	?

Given data

WFR	PT INR Day #5	PT INR Day #6	PT INR Day #7	PT INR Day #8	.	.
1.0 mg	.	.	.	.	.	.
1.5 mg	.	.	.	.	.	.
2.0 mg	.	.	.	.	.	.
2.5 mg	.	.	.	.	.	.
3.0 mg	.	.	.	.	.	.
3.5 mg	.	.	.	.	.	.
4.0 mg	.	.	.	.	.	.
.	.	.	.	.	.	.
.	.	.	.	.	.	.

Individualized prediction table

For example, we have given data (real values), and want to fill up the estimated PT INRs for repeated administration of daily 2.5 mg warfarin.

WFR virtual dose	PT INR Day #5	PT INR Day #6	PT INR Day #7	PT INR Day #8	.	.
2.0 mg	.	.	.	.	.	.
2.5 mg		.	.	.	.	.
3.0 mg	.	.	.	.	.	.
3.5 mg	.	.	.	.	.	.
.	.	.	.	.	.	.

Real values

	Day #1	Day #2	Day #3	Day #4	Day #5	Day #6	Day #7
PT INR	1.14	1.69	1.98	2.11			
WFR	4	4	3				

Sex	Age	Weight	Height	BSA
F	68	53	160	1.53

The seven numbers (PT INR day #1-4 and warfarin dose #1-3) and a virtual dose (2.5 mg) is entered into the algorithm, and it estimates PT INR day #5.

WFR virtual dose	PT INR Day #5	PT INR Day #6	PT INR Day #7	PT INR Day #8	.	.
2.0 mg	.	.	.	.	.	.
2.5 mg	2.04	.	.	.	.	.
3.0 mg	.	.	.	.	.	.
3.5 mg	.	.	.	.	.	.
.	.	.	.	.	.	.

Real values

	Day #1	Day #2	Day #3	Day #4	Day #5	Day #6	Day #7
PT INR	1.14	1.69	1.98	2.11	2.04		
WFR	4	4	3	2.5			

1st virtual dose

1st predicted value

Sex	Age	Weight	Height	BSA
F	68	53	160	1.53

The next seven numbers (PT INR day #2-5 and warfarin dose #2-4) and a virtual dose (2.5 mg) is entered into the algorithm, and it estimates PT INR day #6.

WFR virtual dose	PT INR Day #5	PT INR Day #6	PT INR Day #7	PT INR Day #8	.	.
2.0 mg	.	.	.	.	.	.
2.5 mg	2.04	2.00	.	.	.	.
3.0 mg	.	.	.	.	.	.
3.5 mg	.	.	.	.	.	.
.	.	.	.	.	.	.

Data succeeded from previous day

	Day #1	Day #2	Day #3	Day #4	Day #5	Day #6	Day #7
PT INR	1.14	1.69	1.98	2.11	2.04	2.00	
WFR	4	4	3	2.5	2.5		

2nd virtual dose

2nd predicted value

Sex	Age	Weight	Height	BSA
F	68	53	160	1.53

The next seven numbers (PT INR day #3-6 and warfarin dose #3-5) and a virtual dose (2.5 mg) is entered into the algorithm, and it estimates PT INR day #7.

WFR virtual dose	PT INR Day #5	PT INR Day #6	PT INR Day #7	PT INR Day #8	.	.
2.0 mg	.	.	.	.	.	.
2.5 mg	2.04	2.00	1.96	.	.	.
3.0 mg	.	.	.	.	.	.
3.5 mg	.	.	.	.	.	.
.	.	.	.	.	.	.

Data succeeded from previous day

	Day #1	Day #2	Day #3	Day #4	Day #5	Day #6	Day #7
PT INR	1.14	1.69	1.98	2.11	2.04	2.00	1.96
WFR	4	4	3	2.5	2.5	2.5	

3rd virtual dose

3rd predicted value

Sex	Age	Weight	Height	BSA
F	68	53	160	1.53

In this manner, the whole table can be filled up with estimated PT INRs.

Sex	Age	Weight	Height	BSA
F	68	53	160	1.53

	Day #1	Day #2	Day #3	Day #4
PT INR	1.14	1.69	1.98	2.11
WFR	4 mg	4 mg	3 mg	?

Given data

WFR	PT INR Day #5	PT INR Day #6	PT INR Day #7	PT INR Day #8	.	.
1.0 mg	1.97	1.65	1.44	1.38	.	.
1.5 mg	1.99	1.78	1.60	1.53	.	.
2.0 mg	2.01	1.89	1.79	1.77	.	.
2.5 mg	2.04	2.00	1.96	1.97	.	.
3.0 mg	2.07	2.11	2.14	2.16	.	.
3.5 mg	2.09	2.24	2.34	2.40	.	.
4.0 mg	2.11	2.36	2.55	2.65	.	.
.	.	.	.	.	.	.
.	.	.	.	.	.	.

Individualized prediction table

Section 5. Comparison of performance in predicting the 8th-day PT INR

S5. Preparing a comparable dataset from the test dataset.

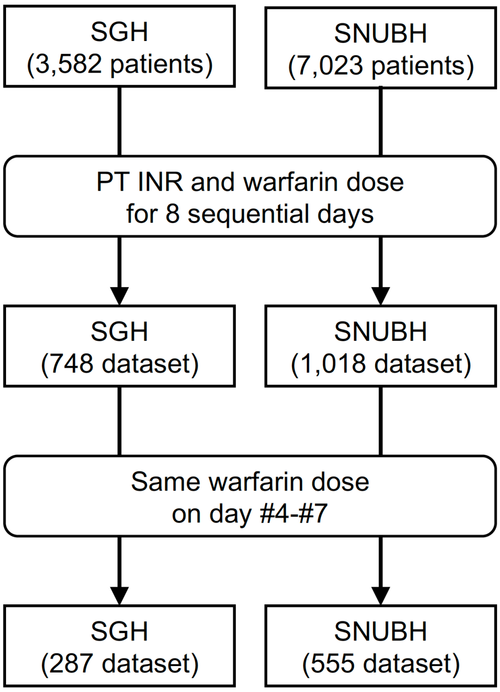

Section 6. Model performance in each hospital

S6. The performance of PT INR Day #5 prediction model in each hospital dataset

Accuracy	SGH dataset (n = 5,719)	SNUBH dataset (n = 6,954)	Total (n = 12,673)
**Within 0.2 of the actual value**	4,363 (76.3%)	4,443 (63.9%)	1,320 (66.0%)
**Within 0.25 of the actual value**	4,788 (83.7%)	5,079 (73.0%)	1,493 (74.7%)
**Within 0.3 of the actual value**	5,086 (88.9%)	5,564 (80.0%)	1,637 (81.9%)
**More 0.5 away from the actual value**	159 (2.78%)	446 (6.41%)	93 (4.7%)
**More than 1.0 away from the actual value**	17 (0.30%)	27 (0.39%)	7 (0.4%)
**| predicted value – actual value | (absolute error)**	0.144 ± 0.156	0.191 ± 0.179	0.173 ± 0.174

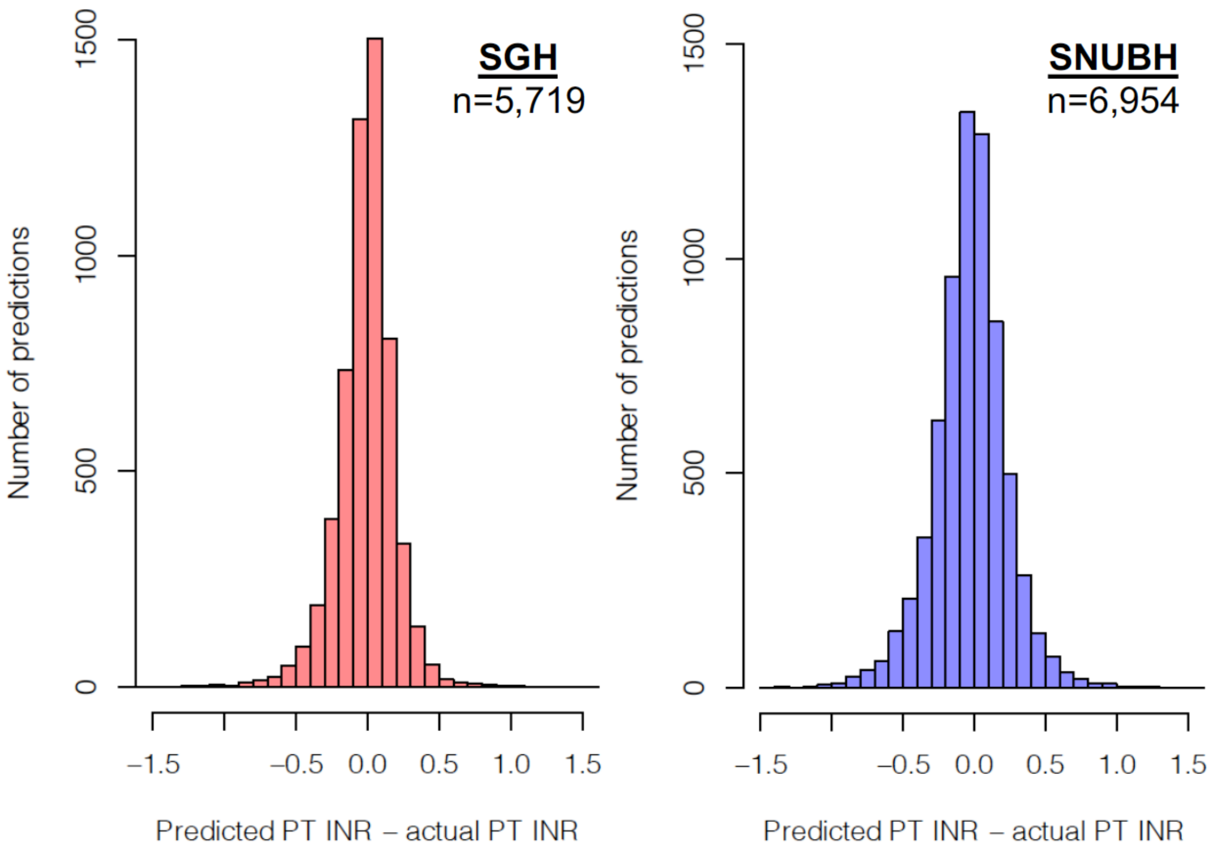

Supplement: Supplementary file 1 — Supplementary Information. [file 41598_2021_94305_MOESM1_ESM.pdf]
